# Structural insights into eukaryotic DNA replication

**DOI:** 10.3389/fmicb.2014.00444

**Published:** 2014-08-25

**Authors:** Sylvie Doublié, Karl E. Zahn

**Affiliations:** Department of Microbiology and Molecular Genetics, University of VermontBurlington, VT, USA

**Keywords:** DNA polymerase, B family, eukaryotic replication, fidelity, proofreading

## Abstract

Three DNA polymerases of the B family function at the replication fork in eukaryotic cells: DNA polymerases α, δ, and ε. DNA polymerase α, an heterotetramer composed of two primase subunits and two polymerase subunits, initiates replication. DNA polymerases δ and ε elongate the primers generated by pol α. The DNA polymerase from bacteriophage RB69 has served as a model for eukaryotic B family polymerases for some time. The recent crystal structures of pol δ, α, and ε revealed similarities but also a number of unexpected differences between the eukaryotic polymerases and their bacteriophage counterpart, and also among the three yeast polymerases. This review will focus on their shared structural elements as well as the features that are unique to each of these polymerases.

## Introduction

Replication in the nucleus of eukaryotic cells employs three DNA polymerases: polymerase α, δ, and ε (Hubscher et al., 2002; Pavlov et al., 2006b; Kunkel and Burgers, 2008; Loeb and Monnat, 2008; Burgers, 2009; Pavlov and Shcherbakova, 2010; Lange et al., 2011). DNA synthesis is directional and proceeds from 5′ to 3′, where nucleophilic attack on the α phosphate of a nucleotide by the 3′OH of a primer results in the incorporation of a nucleoside monophosphate and release of pyrophosphate (Steitz, 1999). All DNA polymerases require a primer and a free 3′OH to conduct DNA synthesis, and pol α is no exception. Pol α is a heterotetramer composed of two primase subunits and two polymerase subunits. The primase subunits initiate DNA replication by synthesizing short (7–12 ribonucleotides) RNA primers, which are then extended by polymerase α (Pellegrini, 2012). DNA polymerase δ and ε elongate the primers generated by pol α in an accurate and processive manner (Kunkel, 2004, 2011; Pellegrini, 2012). In yeast, DNA polymerase δ has been shown to be essential for DNA synthesis of the lagging strand whereas pol ε appears to mainly function at the leading strand (Pursell et al., 2007; Nick Mcelhinny et al., 2008; Kunkel, 2011; Georgescu et al., 2014). In contrast, in the mitochondria replication is the responsibility of one sole polymerase, DNA polymerase γ (Lee et al., 2009).

DNA polymerases are grouped into seven families (A, B, C, D, X, Y, and RT). In eukaryotes the three nuclear replicative DNA polymerases happen to belong to the B family (Burgers et al., 2001; Patel and Loeb, 2001). There are now crystals structures of all three replicative DNA polymerases from yeast, which allow for the first time a comparison of their shared structural elements as well as a study of their unique features (Swan et al., 2009; Perera et al., 2013; Hogg et al., 2014; Jain et al., 2014a). All three replicative DNA polymerases are multi-subunit enzymes (Table 1) (Johansson and Macneill, 2010; Pavlov and Shcherbakova, 2010; Makarova et al., 2014). The main focus of this review is on their catalytic domain, or subunit A.

**Table 1:** **Eukaryotic DNA polymerases are multi-subunit enzymes**.

**Polymerase**	**Species**		**Function**
**Polymerase α**	***H. sapiens***	***S. cerevisiae***	
Catalytic or A-subunit	POLA1 (p180)	POL1	Catalytic subunit; polymerase activity; inactivated exonuclease
B-subunit	POLA2 (p70)	POL12	Regulatory subunit
Primase small subunit	PRIM1 (p49)	PRI1	Primase
Primase large subunit	PRIM2 (p58)	PRI2	Primase
**Polymerase δ**	***H. sapiens***	***S. cerevisiae***	
Catalytic or A-subunit	POLD1 (p125)	POL3	Catalytic subunit; has both polymerase and exonuclease activity
B-subunit	POLD2 (p50)	POL31	Accessory subunit
C-subunit	POLD3 (p66 or p68)	POL32	Accessory subunit
D-subunit	POLD4 (p12)	–	Accessory subunit
**Polymerase ε**	***H. sapiens***	***S. cerevisiae***	
Catalytic or A-subunit	POLE or POLE1	POL2	Catalytic subunit; has both polymerase and exonuclease activity
B-subunit	POLE2	DPB2	Accessory subunit
C-subunit	POLE3 (p17; CHRAC17)	DPB3	Accessory subunit
D-subunit	POLE4 (p12)	DPB4	Accessory subunit

## Overall structure of B family polymerases

All DNA polymerases share a common polymerase fold, which has been compared to a human right hand, composed of three subdomains: fingers, palm, and thumb (Steitz, 1999; Patel and Loeb, 2001). The palm, a highly conserved fold composed of four antiparallel β strands and two helices, harbors two strictly conserved catalytic aspartates located in motif A, **D**XXLYPS and motif C, DT**D**S (Delarue et al., 1990; Braithwaite and Ito, 1993). This RRM-like fold is shared by a very large group of enzymes, including DNA and RNA polymerases, reverse transcriptases, CRISPR polymerase, and even reverse (3′–5′) transferases such as Thg1 (Anantharaman et al., 2010; Hyde et al., 2010). In contrast, the thumb and fingers subdomains exhibit substantially more structural diversity (Steitz, 1999). The fingers undergo a conformational change upon binding DNA and the correct incoming nucleotide. This movement allows residues in the fingers subdomain to come in contact with the nucleotide in the nascent base pair. The thumb holds the DNA duplex during replication and plays a part in processivity (Doublié and Ellenberger, 1998; Doublié et al., 1999).

Eukaryotic DNA polymerases α, δ, and ε share homology with many archaeal, bacterial, bacteriophage, and viral polymerases (Delarue et al., 1990; Braithwaite and Ito, 1993; Franklin et al., 2001; Firbank et al., 2008; Wang and Yang, 2009). Koonin and collaborators contributed a detailed phylogenetic analysis of archaeal DNA polymerases and their relationship with eukaryotic polymerases in this issue of Frontiers in Microbiology dedicated to polymerases Makarova et al. (2014).

**Figure 1:**
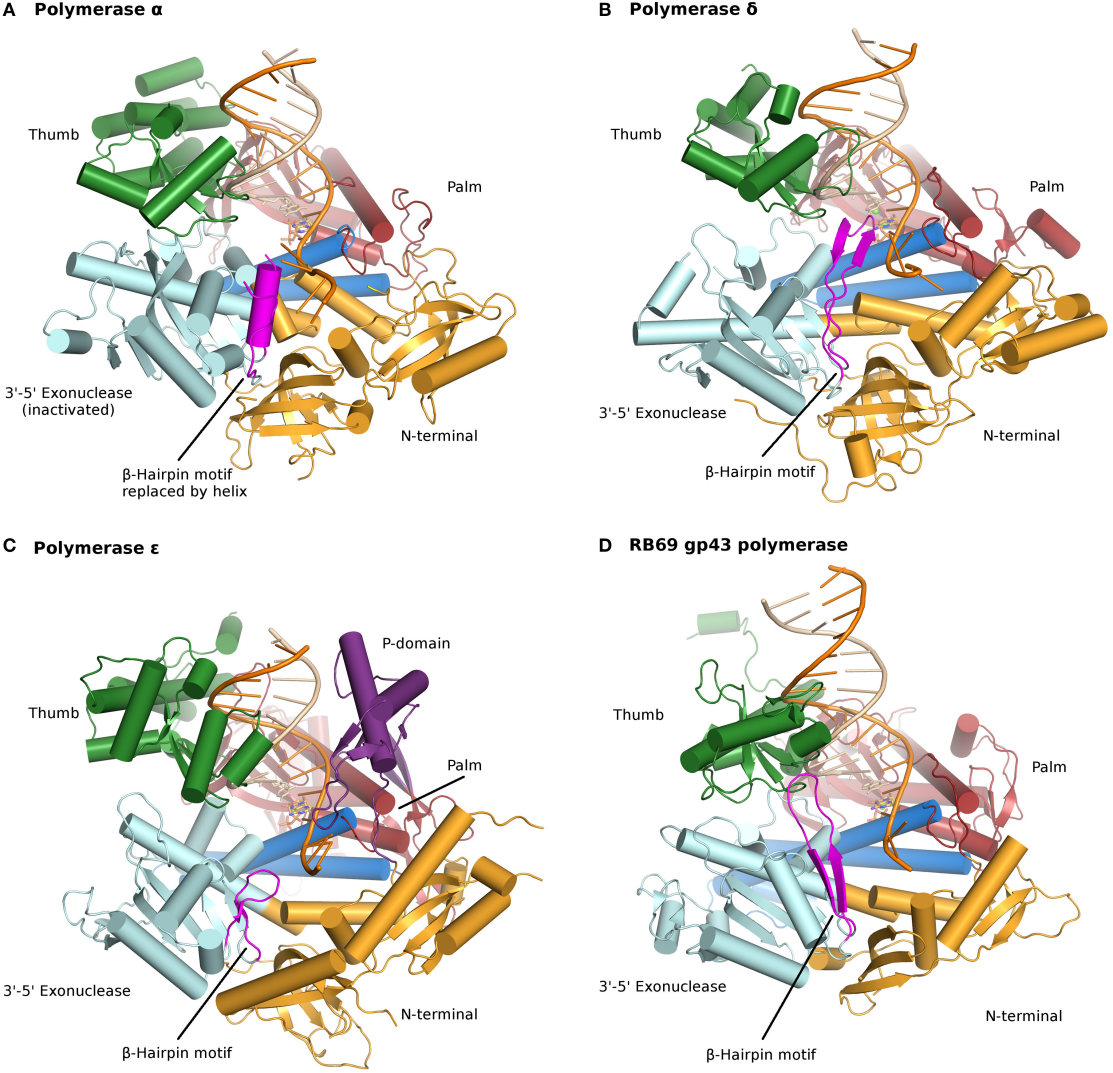
**Ternary complexes of polymerases α, δ, ε, and RB69 gp43 are illustrated from identical orientations for comparison**. The thumb (green) and fingers (dark blue) domains grasp the duplex nucleic acid (primer shown in beige, template in orange) against the palm domain (red). The N-terminal domain appears in gold, adjacent to the 3′–5′ exonuclease domain (cyan). **(A)** Polymerase α (PDBID 4FYD) binds an RNA/DNA hybrid, where the wide, shallow minor groove of A-form DNA is apparent near the thumb. The 3′–5′ exonuclease domain is devoid of activity. A helical region (magenta) in the inactivated exonuclease domain stabilizes the 5′end of the template. **(B)** Polymerase δ (PDBID 3IAY) harbors a large β hairpin motif (magenta), which is important in switching the primer strand from the polymerase active site to the exonuclease active site in the event of proofreading. **(C)** Polymerase ε (PDBID 4M8O) wields a unique P-domain (purple), which endows the polymerase with increased processivity. Interestingly, the β hairpin motif is atrophied in pol ε. **(D)** Conservation of the family B DNA polymerase fold, and domain organization, is evident when the model enzyme from bacteriophage RB69 gp43 (PDBID 2OZS) is viewed along with the three eukaryotic replicative polymerases. The domain delineation for each polymerase is given in Table S1. Figure was made with PyMOL (The PyMOL Molecular Graphics System, Version 1.5.0.4 Schrödinger, LLC.).

All B family polymerases are composed of five subdomains: the fingers, thumb, and palm (described above) constituting the core of the enzyme, as well as an exonuclease domain and an N-terminal domain (NTD) (Franklin et al., 2001; Xia and Konigsberg, 2014) (Figure 1; Table S1). The exonuclease domain carries a 3′–5′ proofreading activity, which removes misincorporated nucleotides. The exonuclease active site is located 40–45 Å away from the polymerase active site. The NTD seems to be devoid of catalytic activity. In pol δ the NTD comprises three motifs: one has a topology resembling an OB fold, a single-stranded DNA binding motif, and another bears an RNA-binding motif (RNA Recognition Motif or RRM) (Swan et al., 2009). In bacteriophage T4, mutations in the NTD decrease expression of the polymerase (Hughes et al., 1987). In RB69 and T4, the gp43 polymerase binds its own messenger RNA, presumably through the NTD and represses translation (Petrov et al., 2002), which does not seem to be the case for pol δ (Swan et al., 2009). New data indicate that the NTD plays a role in polymerase stability and fidelity through its interactions with other domains (Li et al., 2010; Prindle et al., 2013) (see below).

**Figure 2:**
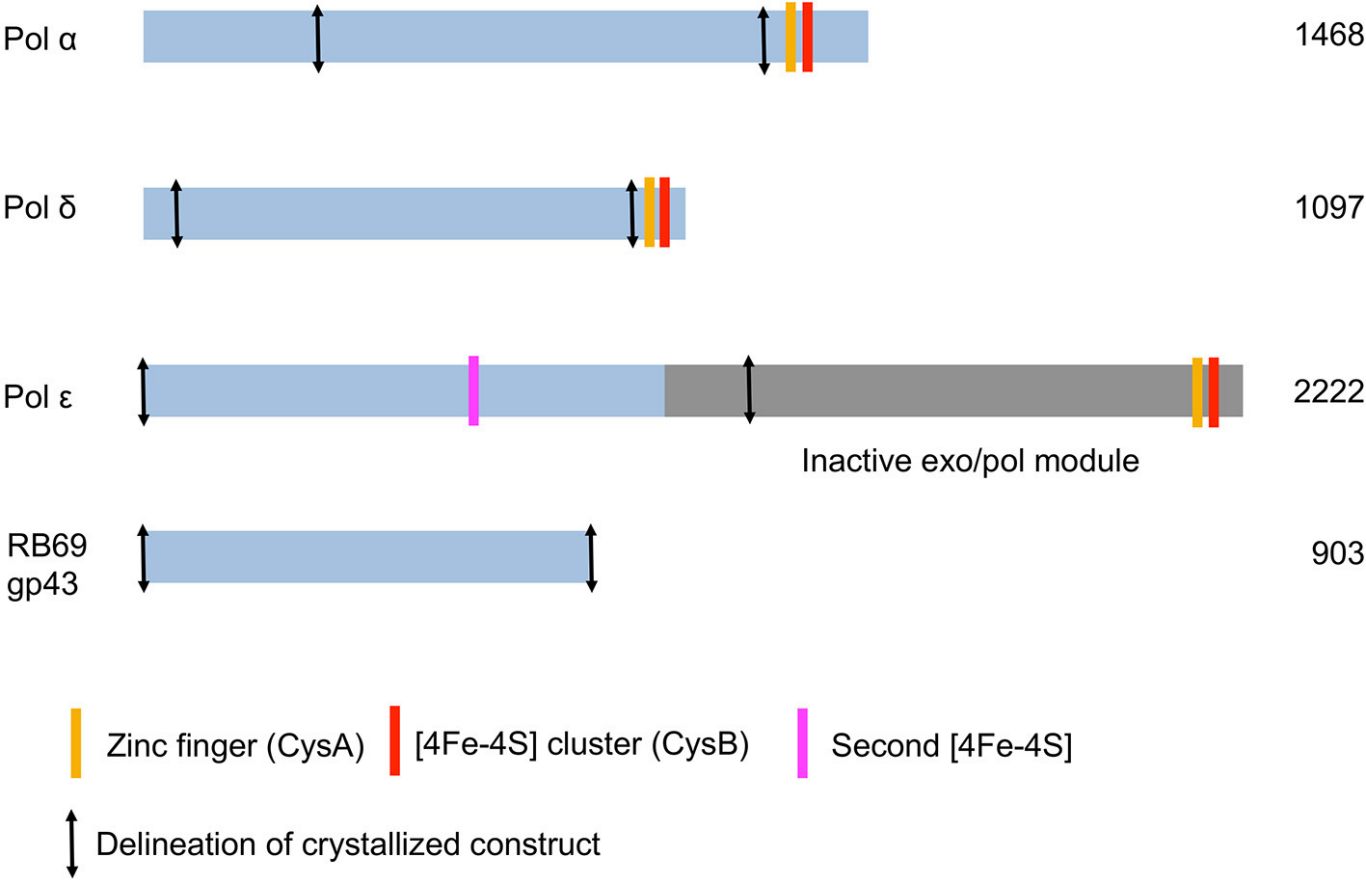
**Schematic diagram of the three *Saccharomyces cerevisiae* replicative DNA polymerases α, δ, and ε**. The DNA polymerase from bacteriophage RB69 is shown for comparison.

All mammalian B family DNA polymerases are known to harbor two cysteine-rich metal binding sites (CysA and CysB) in their C-terminal domain (CTD) (Figure 2). CysA is presumed to be a zinc-binding site whereas CysB is an iron sulfur cluster [4Fe-4S] (Netz et al., 2012). Loss of the [4Fe-4S] cluster in the CTD of yeast pol δ negatively affects interactions with its accessory B-subunit (Sanchez Garcia et al., 2004). The zinc-binding motif was shown to be important for interaction of pol δ with its processivity factor, PCNA (Netz et al., 2012).

## DNA polymerase α

The catalytic subunit of DNA polymerase α is composed of 1468 amino acids (Table 2). The protein construct designed for crystallization was truncated at the N- and C-termini (residues 349–1258) and therefore lacks the CTD and its [4Fe-4S] cluster (Figure 2). The construct was crystallized unliganded, in a binary complex with a DNA/RNA hybrid oligonucleotide, and in a ternary complex with DNA/RNA and incoming nucleotide (Perera et al., 2013) (Figure 1).

**Table 2:** **Compilation of DNA polymerases of the B family of known structure**.

**Polymerase name**	**Yeast gene**	**Conserved aspartates in polymerase active site**	**Conserved carboxylates in exonuclease active site**	**UNIPROT ID**	**Source**	**Iron sulfur clusters?**	**Number of amino acids**	**Protein construct crystallized**	**PDB ID code and reference**
Pol α	POL1	Asp864	N/A	P13382	*S. cerevisiae*	1 [4Fe-4S]	1468 aa	349–1258	4FYD
		Asp998				Cys1348,1353,1367,1372			3.1 Å
									Perera et al., 2013
		Asp996							
Pol δ	POL3	Asp608	Asp321	P15436	*S. cerevisiae*	1 [4Fe-4S]	1097 aa	67–985	3IAY
		Asp764	Glu323			Cys1056,1059,1069,1074			2.0 Å
		Asp762	Asp407						Swan et al., 2009
			Asp520						
Pol ε	POL2	Asp640	Asp290	P21951	*S. cerevisiae*	2 [4Fe-4S]	2222 aa	1-1228	4M8O 2.2 Å
		Asp877	Glu292			Cys665,667,668,763		1-1187	(Hogg et al., 2014)
		Asp875	Asp383			Cys2164,2167,2179,2181			4PTF 2.8 Å
			Asp477						Jain et al., 2014a
RB69 gp43		Asp411	Asp114	Q38087	Bacteriophage RB69	None	903 aa	1-903	1IG9
		Asp623	Glu116						2.6 Å
		Asp621	Asp222						Franklin et al., 2001
			Asp327						
*E. coli* Pol II		Asp419	Asp156	P21189	*Escherichia coli*	None	783 aa	1-780	3K57
		Asp547	Glu158						2.08 Å
		Asp545	Asp229						Wang and Yang, 2009
			Asp315						
Archaeal Tgo		Asp404	Asp141	P56689	*Thermococcus gorgonarius*	None	773 aa	1-757	2VWJ
		Asp542	Glu143						2.78 Å
		Asp540	Asp215						Firbank et al., 2008
			Asp335						

### A mechanism of disengagement of the polymerase

The RNA/DNA oligonucleotide captured in the crystals adopts an A-form conformation, as expected. The thumb domain engages in multiple interactions with the RNA primer, both via hydrophobic contacts and polar interactions (Perera et al., 2013). Experiments in solution have shown that the extension of the RNA primer by pol α is limited to 10–12 nucleotides, which amounts to one turn of a helix. This observation led the authors to suggest a mechanism for termination of primer synthesis by pol α in which loss of specific interactions between the thumb and the RNA would trigger the polymerase to disengage from the DNA/RNA oligonucleotide, and allow a hand off to a replicative polymerase.

### Movements in the palm domain may facilitate translocation of Pol α

Having crystallized the enzyme in three states (apo, binary, and ternary) allowed the authors to overlay all three structural models. Pol α is the only eukaryotic family B DNA polymerase for which all three states were captured in a crystal structure. The structural superposition revealed that, in addition to the well-documented movements of the fingers and thumb subdomains accompanying substrate binding and nucleotidyl transfer, the palm subdomain itself undergoes a structural rearrangement (Perera et al., 2013). The authors propose that the different conformations of the palm domain could facilitate translocation of pol α along and beyond the RNA/DNA duplex. As mentioned above, loss of contacts to the RNA strand is predicted to trigger release of primer, which then becomes available for extension by pol δ or ε.

### A different protein fold in the inactivated exonuclease subdomain

The proofreading activity is abolished in pol α, due to mutations in all four carboxylates (Asp114/Glu116/Asp222/Asp327 in RB69 gp43 correspond to Ser542/Gln544/Tyr644/Asn757 in a structure-based alignment) (Table 2). Moreover, the β-hairpin motif found in most polymerases of the B family (residues 246–267 in RB69 gp43) is replaced by a helical region in pol α (residues 667–676; 681–693) (Hogg et al., 2007). The β hairpin is part of the exonuclease domain and has been shown in T4 and RB69 pols to participate in the partitioning of the DNA primer between the polymerase and the exonuclease active site (Reha-Krantz, 1988; Stocki et al., 1995; Hogg et al., 2007). In the absence of proofreading activity it is not surprising that this motif was not retained in pol α. Residues His 684 and Phe 685 of the helical region in pol α stack with a thymine and guanine base, respectively, at positions -3 and -2 in the unpaired 5′end of the template (Perera et al., 2013). Thus, in pol α the region corresponding to the β-hairpin motif adopts a different fold (helices vs. β strands) and a different function (stabilizing the unpaired region of the template strand rather than facilitating active site switching). Since pol α is devoid of proofreading activity the question arises as to whether the short oligonucleotides are corrected, and if so, by which DNA polymerase. It appears that proofreading of the primers synthesized by pol α is performed by pol δ (Pavlov et al., 2006a).

## DNA polymerase δ

Human pol δ is composed of four subunits whereas *Saccharomyces cerevisiae* has three (Gerik et al., 1998; Liu et al., 2000) (Table 1). In addition to its function in DNA replication pol δ has been shown to play a role in DNA repair and recombination (Hubscher et al., 2002; Lee et al., 2012; Tahirov, 2012). P12, the smallest subunit in human pol δ and also the subunit that is not seen in budding yeast, is degraded in response to DNA damage (Lee et al., 2014). The catalytic subunit of yeast pol δ (POL3) is composed of 1097 residues. The construct used for crystallization comprises residues 67–985 and thus lacks the CTD (Figure 1; Table 2).

### A third metal ion in the polymerase active site

The palm domain contains three conserved carboxylates (Asp608, Asp762, and Asp764). The two catalytic aspartates, Asp608 and Asp764, contact two metal ions (Ca^2+^) in the polymerase active site separated by 3.7 Å. Intriguingly a third metal was observed coordinated by the γ phosphate of the incoming nucleotide and Glu802, with Glu800 in the vicinity. Mutating both glutamates to alanine yielded a polymerase variant with reduced incorporation efficiency for both correct and incorrect nucleotides (Swan et al., 2009). At these amino acid positions, pol α and pol ε also have carboxylate residues (pol δ Glu800/Glu802 correspond to pol α Asp1033/Asp1035, and pol ε Glu945/Asp947). Whether these carboxylates play similar roles in pol α and ε remains to be investigated.

### High fidelity and proofreading

Human pol δ is a high-fidelity polymerase, catalyzing the nucleotidyl transfer reaction with an error frequency of 1 per 22,000 (Schmitt et al., 2009). Proofreading boosts the fidelity of the polymerase by a factor of 10–100 (Mcculloch and Kunkel, 2008; Prindle et al., 2013). Pol δ harbors a polymerase and exonuclease active site, separated by about 45 Å (Swan et al., 2009). DNA polymerases with proofreading activity are able to sense misincorporated nucleotides by contacting the minor groove of base pairs beyond the insertion site. The protein interacts with universal hydrogen bond acceptors at the N3 and O2 positions of purines and pyrimidines, respectively (Seeman et al., 1976; Doublié et al., 1998; Franklin et al., 2001). These hydrogen bond contacts are preserved when the base pair adopts a Watson-Crick geometry and lost in the event of a mismatch. In RB69 gp43, the contacts extend to the first two base pairs beyond the nascent base pair (Franklin et al., 2001; Hogg et al., 2004, 2005). The contacts are much more extensive in pol δ, extending to five base pairs post-insertion (Swan et al., 2009), which could contribute to its high fidelity.

As mentioned above, the β-hairpin segment from the exonuclease domain plays a critical role in the partition of the DNA between polymerization and proofreading sites in T4 and RB69 pols (Stocki et al., 1995; Hogg et al., 2007). In RB69 gp43 the β-hairpin motif adopts different conformations, depending on whether the complex was obtained with undamaged DNA (Franklin et al., 2001; Zahn et al., 2007) or DNA containing a damage (Freisinger et al., 2004; Hogg et al., 2004). It was fully visualized contacting both the primer and template strands in a complex with thymine glycol (Aller et al., 2011). Similarly, the β hairpin in pol δ protrudes into the major groove of the DNA and acts as a wedge between double-stranded DNA and the single-stranded 5′end of the template strand, which is stabilized by two aromatic residues Phe441 and Tyr446 (Figure 1) (Swan et al., 2009). The position of the β hairpin is consistent with a role in active site switching.

### Interdomain contacts and fidelity

Mutations involved in cancer are mostly found in the exonuclease domain of pol δ and ε, emphasizing the critical role of proofreading in lowering the incidence of mutations (Church et al., 2013; Henninger and Pursell, 2014). One mutation in human colorectal cancer cells localizes to the fingers domain, R689W. The analogous mutation in yeast (R696W) results in a mutator phenotype (Daee et al., 2010). A mutation in the vicinity of Arg696 in the highly conserved motif B of the fingers subdomain of yeast pol δ (A699Q) also results in a mutator phenotype. This region of the fingers is in close proximity to the NTD. Mutating Met540 of the NTD to alanine abolishes the mutator phenotype of A699Q, illustrating that interactions between the fingers and the NTD can affect the fidelity of the polymerase (Prindle et al., 2013). Similarly in T4 and RB69 pols the NPL core motif, which involves residues from the N-terminal and palm domains, is in contact with the fingers domain and was shown to stabilize polymerase-DNA complexes (Li et al., 2010).

## DNA polymerase ε

The catalytic subunit of DNA polymerase ε is the product of a very large gene (2222 amino acids in yeast; 2286 in humans), and is only third in size after polymerase ζ (also a member of the B family) and pol θ, a family A polymerase (3130 and 2590 amino acids, respectively, in humans) (Lange et al., 2011; Hogg and Johansson, 2012) (Figure 1; Table 2). Pol ε is twice as large as pol δ and is composed of two tandem polymerase/exonuclease regions. The N-terminal segment harbors both polymerase and proofreading activities whereas the C-terminal segment is inactivated. The two exonuclease-polymerase modules are distantly related (Tahirov et al., 2009). Although the inactivated segment is presumed to play a structural role during replication, two groups were able to crystallize catalytically active pol ε constructs (residues 1–1228; 1–1187) lacking the entire C-terminal module (Hogg et al., 2014; Jain et al., 2014a). Both crystal structures were of a ternary complex of the polymerase, DNA primer/template and incoming nucleotide.

### A novel processivity domain emanating from the palm domain

Pol ε differs from pol δ in that it does not require the DNA sliding clamp PCNA for high processivity (Hogg and Johansson, 2012). The palm domain of pol ε is substantially larger (380 residues) than that of pol α or δ (175 and 203 residues, respectively). The recent pol ε crystal structures revealed that insertions in the palm domain collectively form a new domain consisting of three β strands and two helices (residues 533–555; 682–760) (Hogg et al., 2014; Jain et al., 2014a) (Figure 1; Table S1). Deleting residues 690–751 resulted in a variant with decreased polymerase activity. Moreover, mutating positively charged residues (His748, Arg749, and Lys751) located in the vicinity of the phosphate backbone affected the processivity of the enzyme (Hogg et al., 2014). The extra domain originating from the palm was thus named the processivity or P domain, after its function. The base of the P domain harbors a metal binding site (see below) (Hogg et al., 2014; Jain et al., 2014a,b).

### An iron sulfur cluster within the polymerase domain

Unexpectedly solution studies revealed that the catalytic subunit of yeast polymerase ε itself contains an [4Fe-4S] cluster within its polymerase fold (Jain et al., 2014b), in addition to the [4Fe-4S] cluster in the CTD (Figure 2; Table 2). The second [4Fe-4S] cluster within pol ε suggests that this polymerase may be more sensitive to oxidative stress (Jain et al., 2014b). The crystal structures of pol ε, however, did not reveal a [4Fe-4S] cluster in the polymerase domain (Hogg et al., 2014; Jain et al., 2014a; Zahn and Doublié, 2014). Two of the cysteines residues are disordered in the structural models and the resulting metal binding site appears to bind zinc (Hogg et al., 2014; Jain et al., 2014a). Substitution of a [4Fe-4S] by a non-native zinc in metal-binding proteins is not unusual (Netz et al., 2012) as [4Fe-4S] clusters are labile. Visualizing the [4Fe-4S] within the polymerase domain of pol ε may necessitate anaerobic conditions.

### A short β-hairpin motif in the exonuclease domain

In any DNA polymerase harboring both polymerase and exonuclease activities the bound DNA is in equilibrium between the two active centers (Beechem et al., 1998). The concentration of incoming nucleotide and the presence of a damaged base or mispair are two factors that influence the transfer of DNA from the polymerase activate site to the proofreading active site. Polymerases monitor the minor groove side of the newly formed base pairs and interact with the universal H bond acceptors, O3, and N2, as a way of checking for mismatches (Seeman et al., 1976; Franklin et al., 2001). A unique feature of pol ε is the contact to the major groove side of the nascent base pair via a residue from the exonuclease domain, Tyr431. Further analysis is warranted to elucidate the potential role of this tyrosine in the high fidelity of pol ε.

In pol δ the β-hairpin segment inserts itself in the DNA and acts as a wedge between single-stranded and double-stranded DNA (Swan et al., 2009). In *E. coli* DNA pol II, the insertion of a β barrel shifts the position of the β hairpin in such a way that polymerization is favored over proofreading (Wang and Yang, 2009). This modification presumably allows this polymerase to carry out translesion synthesis extension. Since pol ε is an accurate DNA polymerase the assumption before knowledge of the crystal structure would be that the β hairpin should be closer to that of pol δ than that of *E. coli* Pol II. Surprisingly, the β-hairpin motif in pol ε is truncated, too short to contact the DNA (Figure 1). Which protein motif, then, might be facilitating active site switching upon sensing of a mispair? The P domain is a good candidate, because of its contacts to both primer and template strands; residues from the P domain could sense replication errors and thus may help facilitate active site switching.

## Conclusions

All three eukaryotic replicative DNA polymerases use a common B-family fold, and each polymerase has incorporated modified structural elements which are unique and tailored for each polymerase's specific function (for example, the addition of the processivity domain in pol ε, a processive polymerase that does not use PCNA, or the modified region contacting the 5′end of the template in pol α, a polymerase devoid of proofreading activity). The fold of B family polymerases is well suited for high-fidelity, replicative polymerases. But surprisingly, it is also used by translesion polymerases. Eukaryotic pol ζ (or REV3L) is a 353 kDa polymerase which functions in translesion synthesis and appears to suppress tumorigenesis (Wittschieben et al., 2010; Lange et al., 2011; Zahn et al., 2011; Hogg and Johansson, 2012; Sharma et al., 2013). The structure of *E. coli* Pol II revealed modifications in the NTD which affect the position of the β hairpin of the exonuclease domain, and thus partitioning of the DNA between the polymerization and proofreading sites (Wang and Yang, 2009). The structure of pol ζ may reveal similar adjustments, which alter the fold employed by high-fidelity, replicative polymerases to render the enzyme less faithful and able to perform translesion synthesis.

### Conflict of interest statement

The authors declare that the research was conducted in the absence of any commercial or financial relationships that could be construed as a potential conflict of interest.
